# An Ensemble Approach to Predict Schizophrenia Using Protein Data in the N-methyl-D-Aspartate Receptor (NMDAR) and Tryptophan Catabolic Pathways

**DOI:** 10.3389/fbioe.2020.00569

**Published:** 2020-06-04

**Authors:** Eugene Lin, Chieh-Hsin Lin, Chung-Chieh Hung, Hsien-Yuan Lane

**Affiliations:** ^1^Department of Biostatistics, University of Washington, Seattle, WA, United States; ^2^Department of Electrical & Computer Engineering, University of Washington, Seattle, WA, United States; ^3^Graduate Institute of Biomedical Sciences, China Medical University, Taichung, Taiwan; ^4^Department of Psychiatry, Kaohsiung Chang Gung Memorial Hospital, Chang Gung University College of Medicine, Kaohsiung, Taiwan; ^5^School of Medicine, Chang Gung University, Taoyuan, Taiwan; ^6^Department of Psychiatry, China Medical University Hospital, Taichung, Taiwan; ^7^Brain Disease Research Center, China Medical University Hospital, Taichung, Taiwan; ^8^Department of Psychology, College of Medical and Health Sciences, Asia University, Taichung, Taiwan

**Keywords:** ensemble boosting, multilayer feedforward neural networks, N-methyl-D-aspartate receptor, precision psychiatry, schizophrenia

## Abstract

In the wake of recent advances in artificial intelligence research, precision psychiatry using machine learning techniques represents a new paradigm. The D-amino acid oxidase (DAO) protein and its interaction partner, the D-amino acid oxidase activator (DAOA, also known as G72) protein, have been implicated as two key proteins in the N-methyl-D-aspartate receptor (NMDAR) pathway for schizophrenia. Another potential biomarker in regard to the etiology of schizophrenia is melatonin in the tryptophan catabolic pathway. To develop an ensemble boosting framework with random undersampling for determining disease status of schizophrenia, we established a prediction approach resulting from the analysis of genomic and demographic variables such as DAO levels, G72 levels, melatonin levels, age, and gender of 355 schizophrenia patients and 86 unrelated healthy individuals in the Taiwanese population. We compared our ensemble boosting framework with other state-of-the-art algorithms such as support vector machine, multilayer feedforward neural networks, logistic regression, random forests, naive Bayes, and C4.5 decision tree. The analysis revealed that the ensemble boosting model with random undersampling [area under the receiver operating characteristic curve (AUC) = 0.9242 ± 0.0652; sensitivity = 0.8580 ± 0.0770; specificity = 0.8594 ± 0.0760] performed maximally among predictive models to infer the complicated relationship between schizophrenia disease status and biomarkers. In addition, we identified a causal link between DAO and G72 protein levels in influencing schizophrenia disease status. The study indicates that the ensemble boosting framework with random undersampling may provide a suitable method to establish a tool for distinguishing schizophrenia patients from healthy controls using molecules in the NMDAR and tryptophan catabolic pathways.

## Introduction

Precision psychiatry, an emerging interdisciplinary paradigm of psychiatry and precision medicine, is progressing into the cornerstone of public health practice (Katsanis et al., 2008; Snyderman, 2012). In terms of diagnostic and therapeutic decisions, precision psychiatry is tailored to the specific patient with psychiatric disorders (Katsanis et al., 2008; Snyderman, 2012). More generally, multiple data types such as genomics and protein data are integrated with state-of-the-art artificial intelligence and machine learning algorithms. Thereby, these integrated frameworks are able to correspondingly learn to provide proper clinical decisions during nearly every stage of patient care in an individual manner, such as diagnosis and treatment of psychiatric disorders (Lin and Chen, 2008a; Lane et al., 2012; Lin and Lane, 2015, 2017). For example, a recent study utilized machine learning models to optimize prediction of antidepressant treatment outcome in patients with major depressive disorder by using genetic and clinical datasets (Lin et al., 2018a).

The N-methyl-D-aspartate receptor (NMDAR) pathway has been a focus of attention in schizophrenia research. The D-amino acid oxidase (DAO) protein and its putative activator, the D-amino acid oxidase activator (DAOA, also known as G72) protein, are two proteins in the NMDAR pathway. *In vitro* studies reported that the G72 protein activates and binds to the DAO protein (Chumakov et al., 2002; Sacchi et al., 2008). Next, the DAO protein in turn oxidizes D-amino acids such as D-serine, an agonist of NMDAR (Chumakov et al., 2002; Sacchi et al., 2008). It has been hypothesized that patients who over-yield the G72 protein may reduce the NMDAR activities, thereby inclining them to schizophrenia (Hashimoto et al., 2003; Lin et al., 2014; Lin and Lane, 2019). Furthermore, it has been suggested that plasma G72 protein levels are notably higher in patients with schizophrenia than in healthy individuals (Lin et al., 2014). Moreover, it has been indicated that the agonist activities in the NMDAR pathway possess appropriate importance in developing novel drug targets for treatment of schizophrenia (Coyle et al., 2003; Goff, 2012; Javitt, 2012; Moghaddam and Javitt, 2012; Ermilov et al., 2013; Lane et al., 2013; Lin et al., 2017a, 2018; Chang et al., 2019). To distinguish healthy individuals from patients with schizophrenia, a previous study also utilized machine learning algorithms (such as logistic regression, naive Bayes, and C4.5 decision tree) to construct predictive models by using the G72 protein and genetic variants (Lin et al., 2018b).

Melatonin, which has an impact on the tryptophan catabolic pathway, is another probable factor with respect to the developmental etiology of schizophrenia (Anderson and Maes, 2012). It is proposed that melatonin plays a role as a biomarker of schizophrenia although the findings were controversial (Morera-Fumero and Abreu-Gonzalez, 2013). It has been reported that plasma melatonin levels were higher, lower, or similar in patients with schizophrenia as compared to healthy controls (Morera-Fumero and Abreu-Gonzalez, 2013). Schizophrenia is also linked with both circadian and metabolic disorders, which are modulated by melatonin (Wulff et al., 2012).

Here, in order to distinguish schizophrenia patients from healthy controls in the Taiwanese population, we employed an ensemble boosting algorithm to build predictive models of schizophrenia disease status by using DAO and G72 protein levels in the NMDAR pathway as well as by using melatonin levels in the tryptophan catabolic pathway. To deal with imbalanced data, we also utilized the random undersampling method at the data level (Galar et al., 2011). To the best of our knowledge, no previous studies have been performed to evaluate predictive models for schizophrenia disease status by using ensemble boosting techniques with random undersampling. We selected the ensemble boosting algorithms because these algorithms are regularly applied to solve complex problems in classification and predictive modeling owing to their superiority in reduction of overfitting, consistency, robust prediction, and better generalization (Yang et al., 2010; Galar et al., 2011; Zhang et al., 2019). This study directly compared the performance of the ensemble boosting models to widely used machine learning algorithms, including support vector machine (SVM), multi-layer feedforward neural networks (MFNNs), logistic regression, random forests, naive Bayes, and C4.5 decision tree. Our analysis demonstrated that our ensemble boosting approach with random undersampling led to better performance.

## Materials and Methods

### Study Population

The study cohort consisted of 355 schizophrenia patients and 86 unrelated healthy controls, who were recruited from the China Medical University Hospital in Taiwan. In this study, both schizophrenia patients and healthy controls were aged 18–65 years, were healthy in the neurological and physical conditions, and had obtained normal laboratory assessments (such as blood routine and biochemical tests). Details of the diagnosis of schizophrenia were published previously (Lin et al., 2014). Briefly, the research psychiatrists evaluated both patients and healthy volunteers by using the Structured Clinical Interview for DSM-IV (SCID) for diagnosis (Lin et al., 2014).

After presenting a complete description of this study to the subjects, we obtained written informed consents in line with the institutional review board guidelines. This study was approved by the institutional review board of the China Medical University Hospital in Taiwan and was conducted in accordance with the Declaration of Helsinki.

### Laboratory Assessments

Plasma G72 protein expression levels were measured by western blotting (Lin et al., 2014). Shortly after 10 mL of blood was collected into EDTA-containing blood collection tubes by using sterile techniques, we processed the blood specimens shortly by using centrifugation at 500 g. After centrifugation, we directly dissected plasma and rapidly stored it at −80°C until western blotting. For western blotting, we depleted 100 μL plasma by using ProteoPrep^®^ Blue Albumin and IgG Depletion Kit. All western blot experiments were repeated for two times.

DAO levels in the serum were measured using commercially available enzyme-linked immunosorbent assay (ELISA) kits according to the manufacture’s recommended protocol (Cloud-Clone Corp, Houston, TX, United States). The detailed method has been described elsewhere (Lin et al., 2017b).

Melatonin protein concentrations were measured using commercially available enzyme-linked immunosorbent assay (ELISA) kits according to the manufacture’s recommended protocol (MyBioSource, San Diego, CA, United States). Briefly, 100 μL plasma samples and the standard were added to each well of a 96-well plate. The solutions were incubated for 2 h at 37°C. The liquid was then removed. 100 μL Biotin-antibody (1×) was added to each well and incubated for 1 h at 37°C. Each well was washed with buffer for three times. 100 μL HRP-avidin (1×) was added to each well and incubated for 1 h at 37°C. Each well was washed with buffer for five times and then incubated with 90 μL substrate solution for 15–30 min at 37°C with the protection from light. 50 μL stop solution was added to each well, and mixed thoroughly. A Benchmark Plus Microplate Reader (Bio-Rad) was used to read the optical density at 450 nm. The concentrations of melatonin in the samples were determined according to a standard curve.

### Statistical Analysis

The Student’s *t*-test was conducted to measure the difference in the means of two continuous variables (Lin et al., 2019). We performed the chi-square test for categorical data. The Kruskal-Wallis test was used to determine if there is statistically significant difference between schizophrenia patients and healthy controls on DAO, G72, and melatonin levels. Furthermore, we utilized multivariable logistic regression analysis to assess causal links between DAO, G72, and melatonin levels with adjustment for age and gender. The criterion for significance was set at *P* < 0.05 for all tests. Data are presented as the mean ± standard deviation.

### Ensemble Boosting Predictive Models

We employed a key ensemble boosting technique called LogitBoost (Friedman et al., 2000) and utilized the Waikato Environment for Knowledge Analysis (WEKA) software (which is available from https://www.cs.waikato.ac.nz/ml/weka/) (Witten et al., 2005) to carry out the predictive ensemble framework. All the experiments were conducted on a computer with Intel (R) Core (TM) i5-4210U, 4 GB RAM, and Windows 7.

**FIGURE 1:**
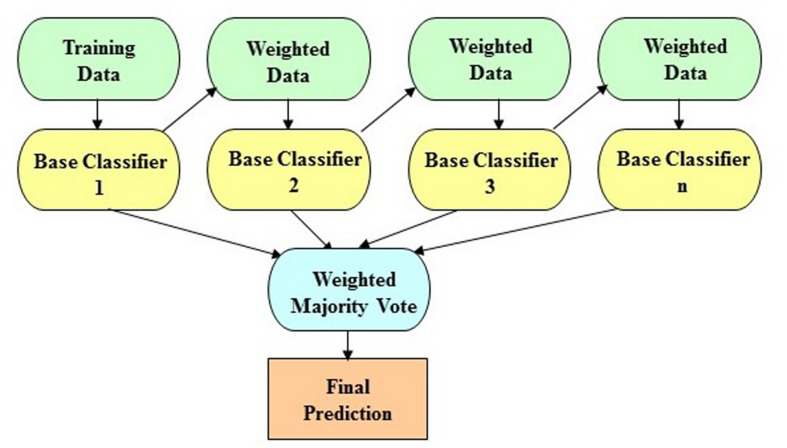
The schematic illustration of the ensemble boosting method. The idea of the ensemble boosting approach is to train weak/base classifiers sequentially in a way that each classifier tries to correct its predecessor. A higher weight is assigned to samples that were incorrectly classified by earlier rounds. That is, week/base classifiers are produced in sequence based on a weighted version of the data during the training phase. The final classification prediction is then produced by a weighted majority vote.

The LogitBoost algorithm is an ensemble boosting approach, which combines the performance of many weak classifiers (also referred to as base classifiers) to achieve a robust classifier with higher accuracy. Figure 1 shows the illustrative diagram of the ensemble boosting method. The LogitBoost algorithm utilizes a binomial log-likelihood method that changes the classification error linearly so that LogitBoost tends to be robust in handling outliers and noisy data. The base classifier we employed is a decision stump, which is a one-level decision tree (that is, a decision tree with a root node and two leaf nodes). Here, we used the default parameters of WEKA, such as 1.0 for the shrinkage parameter, 100 for the batch size, 3.0 for the Z max threshold, and 10 for the number of iterations.

Furthermore, we utilized a random undersampling technique which eliminates instances in the majority class to balance class distribution (Galar et al., 2011). We further combined the LogitBoost algorithm with the random undersampling technique.

### Machine Learning Algorithms for Benchmarking

For the benchmarking task in the present study, we utilized six state-of-the-art machine learning algorithms including SVM, MFNNs, logistic regression, random forests, naive Bayes, and C4.5 decision tree to compare with the ensemble boosting model. We carried out the analyses for these six machine learning algorithms using the WEKA software (Witten et al., 2005) and a computer with Intel (R) Core (TM) i5-4210U, 4 GB RAM, and Windows 7.

The SVM algorithm (Vapnik, 2013) is a popular technique for pattern recognition and classification. Given a training set of instance-label pairs, the SVM algorithm leverages a kernel function to map the training vectors into a higher dimensional space (Lin and Hwang, 2008b; Vapnik, 2013). In this higher dimensional space, the SVM algorithm then finds a linear separating hyperplane with the maximal margin. In this study, we used the Pearson VII function-based universal kernel (Üstün et al., 2006) with the omega value of 1.0 and the sigma value of 0.5.

An MFNN framework consists of one input layer, one or multiple hidden layers, and one output layer, where connections among neuron structures consist of no directed cycles (Bishop, 1995). In the learning period of the MFNN framework, the back-propagation algorithm (Rumelhart et al., 1996) is leveraged for the learning strategy. In the retrieving period, the MFNN framework repeats via all the structures to perform the retrieval process at the output panel in keeping with the inputs of test patterns (Kung and Hwang, 1998).

We used the logistic regression model, the standard method for classification problems in clinical applications (Witten et al., 2005), as a basis for comparison. In addition, we employed the naive Bayes model that assumes the presence or absence of a particular feature is unrelated to the presence or absence of any other feature (Witten et al., 2005). The naive Bayes model calculates the probability that a given instance belongs to a certain class (that is, “schizophrenia patient” or “healthy control” in this study) by using the Bayes’ theorem.

The random forests model is an ensemble learning method that composes a collection of decision trees during training and yields the class that is the mode of the classes among the individual trees (Breiman, 2001). Here, we used the default parameters of WEKA for the random forests model; for example, 100 for the batch size and 100 for the number of iterations.

The C4.5 decision tree model builds decision trees top-down and prunes them using the concept of information entropy (Witten et al., 2005). First, the tree is constructed by finding the root node (for example, protein level) that is most discriminative one for differentiating “schizophrenia patient” from “healthy control.” Then, the best single feature test is decided by the information gain and by choosing a feature (for example, protein level) to split the data into subsets. Here, we used the default parameters of WEKA, such as 0.25 for the confidence factor and 2 for the minimum number of instances per leaf node (Huang et al., 2009).

### Evaluation of the Predictive Performance

In this study, we utilized the receiver operating characteristic (ROC) methodology and determined the area under the ROC curve (AUC) to assess the performance of predictive models (Linden, 2006; Lin and Hwang, 2008b; Huang et al., 2009). The better the prediction model, the higher the AUC (Linden, 2006; Huang et al., 2009). In additional, we calculated sensitivity (that is, the proportion of correctly predicted responders of all tested responders) as:

Sensitivity=True⁢Positive/(True⁢Positive+False⁢Negative)

and specificity (that is, the proportion of correctly predicted non-responders of all the tested non-responders) as:

Specificity=True⁢Negative/(True⁢Negative+False⁢Positive).

Moreover, we utilized the repeated 10-fold cross-validation method and leave-one-out cross-validation method to examine the generalization of predictive models (Huang et al., 2009; Lin and Hsu, 2009).

## Results

### The Study Cohort in the Taiwanese Population

The participants included 355 schizophrenia patients and 86 unrelated healthy individuals in the Taiwanese population. As shown in Table 1, there was no significant difference in gender (*P* = 0.101) and age (*P* = 0.136) distributions between the two groups. The mean age (39.6 ± 10.0 years) of schizophrenia patients was older than that of healthy controls (37.8 ± 12.2 years). The mean level of DAO protein in the plasma of schizophrenia patients was considerably higher than that of healthy controls (37.64 ± 14.18 ng/mL vs. 28.03 ± 9.84 ng/mL; *P* = 5.55 × 10^–9^) (Table 1). In addition, the mean level of G72 protein in the plasma of schizophrenia patients was markedly higher than that of healthy controls (3.24 ± 1.80 ng/μL vs. 1.68 ± 0.81 ng/μL; *P* = 4.71 × 10^–14^) (Table 1). Moreover, the mean level of melatonin in the plasma of schizophrenia patients was notably higher than that of healthy controls (89.89 ± 46.07 pg/mL vs. 60.04 ± 42.72 pg/mL; *P* = 9.75 × 10^–7^) (Table 1).

**TABLE 1:** Demographic characteristics of schizophrenia patients and healthy individuals.

Characteristic	Schizophrenia patients	Healthy individuals	*P*-value^a^
No. of subjects (n)	355	86	
Gender (male)%	61.9%	52.3%	0.101
Age (year)	39.6 ± 10.0	37.8 ± 12.2	0.136
DAO level (ng/mL)	37.64 ± 14.18	28.03 ± 9.84	5.55 × 10^–9^
G72 level (ng/μL)	3.24 ± 1.80	1.68 ± 0.81	4.71 × 10^–14^
Melatonin level (pg/mL)	89.89 ± 46.07	60.04 ± 42.72	9.75 × 10^–7^

*^*a*^Chi-square test for the categorical data; Student’s t-test for continuous variables. Data are presented as mean ± standard deviation. DAO, D-amino acid oxidase; G72 (also known as DAOA), D-amino acid oxidase activator.*

The significant Kruskal-Wallis test was shown for DAO, G72, and melatonin levels (*P* = 3.12 × 10^–9^, 2.2 × 10^–16^, and 3.35 × 10^–6^, respectively) between schizophrenia patients and healthy controls. Supplementary Figure S1 shows the distribution charts of three features (such as DAO, G72, and melatonin levels) and other variables for schizophrenia patients and healthy controls. The distribution charts are grouped separately by two subsets, namely schizophrenia patients (shown in the red color) and healthy controls (shown in the blue color). As shown in Supplementary Figure S1, the number of schizophrenia patients was much larger than the number of healthy controls.

### Predictive Models for Schizophrenia Disease Status

In this study, we used five biomarkers including DAO levels, G72 levels, melatonin levels, age, and gender to build the predictive models for differentiating schizophrenia patients from healthy individuals by employing the ensemble boosting framework. Table 2 summarizes the results of repeated 10-fold cross-validation experiments by ensemble boosting (with random undersampling), SVM, MFNNs, logistic regression, random forests, naive Bayes, and C4.5 decision tree using five biomarkers. To measure the performance of prediction models, we used the ROC methodology and calculated the AUC, sensitivity, and specificity for these predictive models using five biomarkers.

**TABLE 2:** The results of repeated 10-fold cross-validation experiments for differentiating schizophrenia patients from healthy individuals using ensemble boosting with random undersampling, ensemble boosting, SVM, MFNNs, logistic regression, random forests, naive Bayes, and C4.5 decision tree with biomarkers such as DAO protein levels, G72 protein levels, melatonin protein levels, age, and gender.

**Algorithm**	**AUC**	**Sensitivity**	**Specificity**	**Number of biomarkers**
Ensemble boosting with random undersampling	0.9242 ± 0.0652	0.8580 ± 0.0770	0.8594 ± 0.0760	5
Ensemble boosting	0.9010 ± 0.0464	0.8442 ± 0.0447	0.5803 ± 0.1446	5
SVM	0.6720 ± 0.0837	0.8461 ± 0.0393	0.4979 ± 0.1364	5
MFNN with 1 hidden layer	0.8920 ± 0.0463	0.8343 ± 0.0457	0.5816 ± 0.1340	5
MFNN with 2 hidden layers	0.8949 ± 0.0455	0.8391 ± 0.0515	0.6121 ± 0.1383	5
MFNN with 3 hidden layers	0.8884 ± 0.0507	0.8359 ± 0.0463	0.6312 ± 0.1454	5
Logistic Regression	0.8677 ± 0.0566	0.8497 ± 0.0566	0.5660 ± 0.1295	5
Random Forests	0.8543 ± 0.0627	0.8229 ± 0.0379	0.4197 ± 0.1213	5
naive Bayes	0.8546 ± 0.0628	0.8320 ± 0.0473	0.6611 ± 0.1411	5
C4.5 decision tree	0.7701 ± 0.0721	0.8306 ± 0.0469	0.4526 ± 0.1272	5

*AUC, the area under the receiver operating characteristic curve; DAO, D-amino acid oxidase; G72 (also known as DAOA), D-amino acid oxidase activator; MFNNs, multilayer feedforward neural networks; SVM, support vector machine. Data are presented as mean ± standard deviation.*

Supplementary Figures S2–S4 show plots of ROC, precision-recall, and sensitivity-specificity curves for ensemble boosting with random undersampling using five biomarkers, respectively. Supplementary Figures S5–S10 show plots of ROC, precision-recall, and sensitivity-specificity curves for ensemble boosting, SVM, MFNNs, logistic regression, random forests, naive Bayes, and C4.5 decision tree using five biomarkers.

As shown in Supplementary Figure S2, the lower left point (0, 0) on the ROC curve represents a false positive rate of 0% (that is, no false positive errors) and a true positive rate of 0% (that is, no true positives), indicating never having a positive classification. On the contrary, the upper right point (1, 1) represents a false positive rate of 100% and a true positive rate of 100%, indicating completely having positive classifications. Furthermore, if we assume that the point (0.1406, 0.858) is on the ROC curve, the point (0.1406, 0.858) shows a false positive rate of 14.06% (or specificity of 0.8594) and a true positive rate of 85.8% (or sensitivity of 0.858).

As shown in Supplementary Figure S3, if we assume that the point (0.858, 0.8546) is on the precision-recall curve, the point (0.858, 0.8546) shows a true positive rate of 85.8% (or recall/sensitivity of 0.858) and a precision value of 85.46%. Additionally, as shown in Supplementary Figure S4, if we assume that the point (0.8594, 0.858) is on the sensitivity-specificity curve, the point (0.8594, 0.858) shows a true negative rate of 85.94% (or specificity of 0.8594) and a true positive rate of 85.8% (or sensitivity of 0.858).

In addition, Supplementary Tables S1–S3 summarize the results of repeated 10-fold cross-validation experiments by ensemble boosting (with random undersampling), SVM, MFNNs, logistic regression, random forests, naive Bayes, and C4.5 decision tree using individual features such as DAO (Supplementary Table S1), G72 (Supplementary Table S2), and melatonin (Supplementary Table S3) levels, respectively.

### Ensemble Boosting Model for Schizophrenia Disease Status

For the ensemble boosting model for forecasting schizophrenia disease status, we performed a series of different datasets using five biomarkers as well as individual features. As indicated in Table 2, the average value of AUC for the ensemble boosting prediction model with random undersampling was 0.9242 ± 0.0652 using five biomarkers including DAO levels, G72 levels, melatonin levels, age, and gender. As indicated in Supplementary Tables S1–S3, the average values of AUC for the ensemble boosting prediction model with random undersampling were 0.6471 ± 0.1062, 0.7314 ± 0.1121, and 0.8462 ± 0.0873 using individual features such as DAO levels, G72 levels, and melatonin levels, respectively.

### Benchmarking

To evaluate the performance of our approach for predictive models for schizophrenia disease status, we compared the ensemble boosting model with other state-of-the-art methods, including SVM, MFNNs, logistic regression, random forests, naive Bayes, and C4.5 decision tree.

For MFNN models for forecasting schizophrenia disease status, we performed a series of different architectures containing 1, 2, and 3 hidden layers. Supplementary Figures S11–S13 show an example of architecture of the MFNN model with 3, 2, and 1 hidden layer(s), respectively. As indicated in Table 2, the average values of AUC for the MFNN prediction models of 1, 2, and 3 hidden layers were 0.8920 ± 0.0463, 0.8949 ± 0.0455, and 0.8884 ± 0.0507, respectively. Supplementary Figures S14–S16 show cost/loss function measurement plots of the MFNN model with 3, 2, and 1 hidden layer(s), respectively. Of all the MFNN prediction models, the MFNN model with 2 hidden layers yielded better performance than the other two models in terms of AUC. Thus, there was no significant improvement in the sensitivity with the increase in hidden layers. Moreover, the specificity was low, indicating that the model provides more false positives. This may have been due to an imbalance in the dataset.

Supplementary Table S4 shows WEKA’s hyper-parameters for training the MFNN models with 1–3 hidden layers. For example, we used the following WEKA’s parameters for training the MFNN model with one hidden layer: the momentum = 0.01, the learning rate = 0.05, the batch size = 100, and the number of epochs = 500.

As shown in Table 2, the ensemble boosting model with random undersampling performed maximally in all cases. The best AUC was 0.9242 ± 0.0652, which was based on the ensemble boosting model with random undersampling (Table 2). Our analysis indicated that the ensemble boosting model with random undersampling was well-suited for predictive models for schizophrenia disease status. Furthermore, the ensemble boosting model with random undersampling performed best in both sensitivity (0.8580 ± 0.0770) and specificity (0.8594 ± 0.0760) (Table 2).

### Leave-One-Out Cross-Validation Experiments

In this study, we also explored the generalization of predictive models using the leave-one-out cross-validation method. Supplementary Table S5 summarizes the results of leave-one-out cross-validation experiments by ensemble boosting (with random undersampling), SVM, MFNNs, logistic regression, random forests, naive Bayes, and C4.5 decision tree using five biomarkers such as DAO levels, G72 levels, melatonin levels, age, and gender. In addition, Supplementary Tables S6–S8 summarize the results of leave-one-out cross-validation experiments by ensemble boosting (with random undersampling), SVM, MFNNs, logistic regression, random forests, naive Bayes, and C4.5 decision tree using individual features such as DAO (Supplementary Table S6), G72 (Supplementary Table S7), and melatonin (Supplementary Table S8) levels, respectively.

As indicated in Supplementary Table S5, the AUC value for the ensemble boosting prediction model with random undersampling was 0.937 using five biomarkers including DAO levels, G72 levels, melatonin levels, age, and gender. As indicated in Supplementary Tables S6–S8, the AUC values for the ensemble boosting prediction model with random undersampling were 0.603, 0.610, and 0.826 using individual features such as DAO levels, G72 levels, and melatonin levels, respectively.

As shown in Supplementary Table S5, the best AUC was 0.937, which was based on the ensemble boosting model with random undersampling using five biomarkers such as DAO levels, G72 levels, melatonin levels, age, and gender. Furthermore, the ensemble boosting model with random undersampling performed best in both sensitivity (0.855) and specificity (0.855) (Supplementary Table S5).

### Causal Links Between Protein Levels

Finally, we assessed causal links among DAO levels, G72 levels, and melatonin levels in predicting schizophrenia disease status with age and sex as covariates. In our analysis, there was a significant causal link involving DAO levels and G72 levels (*P* = 0.0036) in influencing schizophrenia disease status. However, there were no causal links either between DAO levels and melatonin levels or between G72 levels and melatonin levels.

## Discussion

To our knowledge, this is the first study to date to leverage an ensemble boosting approach with random undersampling for building predictive models of schizophrenia disease status among Taiwanese individuals. Moreover, we performed the first study to predict schizophrenia disease status by utilizing protein data in both the NMDAR and tryptophan catabolic pathways. The findings pinpointed that the ensemble boosting model with random undersampling using five biomarkers outperformed other state-of-the-art predictive models in terms of AUC for distinguishing schizophrenia patients from healthy controls. The five biomarkers encompassed DAO levels, G72 levels, melatonin levels, age, and gender. In addition, we found that a significant causal link between DAO and G72 protein levels possessed a strong potential to reflect schizophrenia disease status. By leveraging the molecular data in the NMDAR and tryptophan catabolic pathways, we establish the predictive models of schizophrenia disease status by using the ensemble boosting framework with random undersampling. Our data also suggest that our ensemble boosting models with random undersampling may provide a suitable approach to create predictive models for forecasting schizophrenia disease status with clinically meaningful accuracy. Therefore, the ensemble boosting approach with random undersampling in this study is a proof of concept of a machine learning predictive tool for discriminating schizophrenia patients from healthy individuals.

Remarkably, an intriguing finding was that we further inferred the causal link between DAO and G72 protein levels in influencing schizophrenia disease status. To our knowledge, scanty human studies have been conducted to evaluate causal links between DAO and G72 protein levels. The biological mechanisms of these causal links in schizophrenia disease status remain to be elucidated. In line with our results, an *in vitro* study identified a physical interaction between DAO and G72 proteins using yeast two-hybrid experiments (Chumakov et al., 2002). Moreover, a recent study found a putative correlation between DAO and G72 protein expressions in the brain regions such as the brainstem, cerebellum, amygdala, and thalamus (except for the frontal cortex) by using post-mortem brain samples in normal human subjects (Jagannath et al., 2017).

In this study, the dataset is highly imbalanced because the class of schizophrenia patients is significantly larger in terms of instances than the class of healthy controls. To overcome this limitation, we employed the random undersampling method to balance class distribution. Without random undersampling, the predictive models tend to have lower specificity values. In line with previous findings (Chawla et al., 2004; Galar et al., 2011), we found that the ensemble boosting model with random undersampling is highly suitable for handling class imbalances. It has also been suggested to use more accurate measures such as AUC to evaluate predictive models in the case of class imbalances (Chawla et al., 2004).

Furthermore, it is worthwhile to bring the discussion on the random undersampling method for dealing with the imbalanced data (that is, the bigger number of schizophrenia patients vs. the smaller number of healthy controls) in our study. Due to the imbalanced data, the models without the random undersampling method showed predictions that were clearly biased toward higher sensitivity and lower specificity. For example, without random undersampling, sensitivity was around 80% and specificity was around 50–60% for the models using the combined biomarkers of DAO, G72, and melatonin protein levels (Table 2). On the contrary, ensemble boosting with random undersampling had sensitivity of 85.8% and specificity of 85.94% for the combined biomarkers (Table 2). The models with individual biomarkers were also in the similar situation (Supplementary Tables S1–S3). For instance, without random undersampling, sensitivity was around 80% and specificity was around 40% for the models using individual melatonin protein levels (Supplementary Table S3). On the other hand, ensemble boosting with random undersampling had sensitivity of 77.19% and specificity of 77.44% for melatonin protein levels (Supplementary Table S3). Therefore, predictions were no longer biased toward higher sensitivity and lower specificity by using ensemble boosting with random undersampling. Our improved results demonstrate that the ensemble boosting model with random undersampling provides an effective way to solve the imbalanced data problem in our study.

## Conclusion

In conclusion, we created an ensemble boosting predictive framework with random undersampling for estimating schizophrenia disease status in Taiwanese subjects by using DAO and G72 protein datasets in the NMDAR pathway as well as by using melatonin dataset in the tryptophan catabolic pathway. The analysis indicates that our ensemble boosting framework with random undersampling could contribute a conceivable way to construct predictive algorithms for determining schizophrenia disease status in terms of clinically purposeful performance. Consequently, we would foresee that the findings of this study may be generalized for genomic medicine studies in precision psychiatry to forecast disease status and treatment response for psychiatric disorders. Furthermore, the findings may be potentially adopted to provide molecular diagnostic and prognostic tools in the coming years. It is indispensable to unfold further discoveries into the role of the machine learning predictive framework explored in this study by using replication studies with independent samples.

## Data Availability Statement

The raw data supporting the conclusions of this article will be made available by the authors, without undue reservation, to any qualified researcher.

## Ethics Statement

This study was approved by the Institutional Review Board of China Medical University Hospital, Taiwan and complies with the Declaration of Helsinki. Informed written consent was obtained from all participants.

## Author Contributions

EL, C-HL, and H-YL designed the study and revised the manuscript. C-HL, C-CH, and H-YL conducted the study. EL analyzed the data and drafted the manuscript. All authors provided the final approval of the version to be published.

## Conflict of Interest

The authors declare that the research was conducted in the absence of any commercial or financial relationships that could be construed as a potential conflict of interest.
