# Supplementary material for: An Ensemble Approach to Predict Schizophrenia Using Protein Data in the N-methyl-D-Aspartate Receptor (NMDAR) and Tryptophan Catabolic Pathways
Source: Front Bioeng Biotechnol. 2020 Jun 4;8:569. doi: 10.3389/fbioe.2020.00569 (PMC7287032; doi:10.3389/fbioe.2020.00569)
Supplement: Supplementary file 1 [file Data_Sheet_1.PDF]

Supplementary information for

An ensemble approach to predict schizophrenia using protein data in the N-methyl-D-aspartate receptor (NMDAR) and tryptophan catabolic pathways

Eugene Lin[1,2,3], Chieh-Hsin Lin[3,4,5], Chung-Chieh Hung[6], Hsien-Yuan Lane[3,6,7,8]

[1] Department of Biostatistics, University of Washington, Seattle, WA 98195, USA

[2] Department of Electrical & Computer Engineering, University of Washington, Seattle, WA 98195, USA

[3] Graduate Institute of Biomedical Sciences, China Medical University, Taichung, Taiwan

[4] Department of Psychiatry, Kaohsiung Chang Gung Memorial Hospital, Chang Gung University College of Medicine, Kaohsiung, Taiwan

[5] School of Medicine, Chang Gung University, Taoyuan, Taiwan

[6] Department of Psychiatry, China Medical University Hospital, Taichung, Taiwan

[7] Brain Disease Research Center, China Medical University Hospital, Taichung, Taiwan

[8] Department of Psychology, College of Medical and Health Sciences, Asia University, Taichung

Table S1. The results of repeated 10-fold cross-validation experiments for differentiating schizophrenia patients from healthy individuals using ensemble boosting with random undersampling, ensemble boosting, SVM, MFNNs, logistic regression, random forests, naive Bayes, and C4.5 decision tree with DAO protein levels.

**Algorithm**	**AUC**	**Sensitivity**	**Specificity**	**Number of biomarkers**
Ensemble Boosting with random undersampling	0.6471±0.1062	0.6030±0.0904	0.6025±0.0922	1
Ensemble Boosting	0.6862±0.0829	0.7953±0.0268	0.2124±0.0417	1
SVM	0.5000±0.0000	0.8050±0.0109	0.1950±0.0109	1
MFNN with 1 hidden layer	0.7066±0.0836	0.8050±0.0109	0.1950±0.0109	1
MFNN with 2 hidden layers	0.7066±0.0836	0.8050±0.0109	0.1950±0.0109	1
MFNN with 3 hidden layers	0.7066±0.0836	0.8050±0.0109	0.1950±0.0109	1
Logistic Regression	0.7066±0.0836	0.8046±0.0195	0.2250±0.0477	1
Random Forests	0.5948±0.0883	0.6777±0.0636	0.3217±0.0956	1
naive Bayes	0.6942±0.0874	0.8050±0.0109	0.1950±0.0109	1
C4.5 decision tree	0.5000±0.0000	0.8050±0.0109	0.1950±0.0109	1

AUC = the area under the receiver operating characteristic curve; DAO = D-amino acid oxidase; MFNNs = multilayer feedforward neural networks; SVM = support vector machine.

Data are presented as mean ± standard deviation.

Table S2. The results of repeated 10-fold cross-validation experiments for differentiating schizophrenia patients from healthy individuals using ensemble boosting with random undersampling, ensemble boosting, SVM, MFNNs, logistic regression, random forests, naive Bayes, and C4.5 decision tree with G72 protein levels.

Algorithm	AUC	Sensitivity	Specificity	Number of biomarkers
Ensemble Boosting with random undersampling	0.7314±0.1121	0.7273±0.0941	0.7272±0.0933	1
Ensemble Boosting	0.7838±0.0572	0.8041±0.0313	0.2434±0.0659	1
SVM	0.5000±0.0000	0.8050±0.0109	0.1950±0.0109	1
MFNN with 1 hidden layer	0.8041±0.0640	0.8032±0.0340	0.2878±0.0916	1
MFNN with 2 hidden layers	0.8041±0.0640	0.8025±0.0327	0.2736±0.0934	1
MFNN with 3 hidden layers	0.8041±0.0640	0.8050±0.0109	0.1950±0.0109	1
Logistic Regression	0.8041±0.0640	0.7970±0.0375	0.3341±0.1018	1
Random Forests	0.7335±0.0676	0.7635±0.0571	0.3884±0.1183	1
naive Bayes	0.7844±0.0703	0.8050±0.0109	0.1950±0.0109	1
C4.5 decision tree	0.5000±0.0000	0.8050±0.0109	0.1950±0.0109	1

AUC = the area under the receiver operating characteristic curve; G72 (also known as DAOA) = D-amino acid oxidase activator; MFNNs = multilayer feedforward neural networks; SVM = support vector machine.

Data are presented as mean ± standard deviation.

Table S3. The results of repeated 10-fold cross-validation experiments for differentiating schizophrenia patients from healthy individuals using ensemble boosting with random undersampling, ensemble boosting, SVM, MFNNs, logistic regression, random forests, naive Bayes, and C4.5 decision tree with melatonin protein levels.

Algorithm	AUC	Sensitivity	Specificity	Number of biomarkers
Ensemble Boosting with random undersampling	0.8462±0.0873	0.7719±0.0964	0.7744±0.0943	1
Ensemble Boosting	0.8178±0.0674	0.8197±0.0400	0.3895±0.1248	1
SVM	0.6234±0.0685	0.8324±0.0389	0.4143±0.1100	1
MFNN with 1 hidden layer	0.7902±0.0929	0.8338±0.0427	0.4546±0.1186	1
MFNN with 2 hidden layers	0.7902±0.0929	0.8334±0.0425	0.4536±0.1243	1
MFNN with 3 hidden layers	0.7902±0.0929	0.8308±0.0419	0.4393±0.1343	1
Logistic Regression	0.6711±0.1230	0.8268±0.0261	0.3120±0.0872	1
Random Forests	0.5536±0.1331	0.8002±0.0392	0.3472±0.1025	1
naive Bayes	0.6684±0.1245	0.8050±0.0109	0.1950±0.0109	1
C4.5 decision tree	0.4666±0.0906	0.8066±0.0137	0.2054±0.0355	1

AUC = the area under the receiver operating characteristic curve; MFNNs = multilayer feedforward neural networks; SVM = support vector machine.

Data are presented as mean ± standard deviation.

Table S4. The hyper-parameters for training the MFNN models using the WEKA tool.

Algorithm	Momentum	Learning rate	Batch size	Number of epochs
MFNN with 1 hidden layer	0.01	0.05	100	500
MFNN with 2 hidden layers	0.01	0.05	100	500
MFNN with 3 hidden layers	0.01	0.2	100	500

MFNN = multilayer feedforward neural network; WEKA: The Waikato Environment for Knowledge Analysis.

Table S5. The results of leave-one-out cross-validation experiments for differentiating schizophrenia patients from healthy individuals using ensemble boosting with random undersampling, ensemble boosting, SVM, MFNNs, logistic regression, random forests, naive Bayes, and C4.5 decision tree with biomarkers such as DAO protein levels, G72 protein levels, melatonin protein levels, age, and gender.

**Algorithm**	**AUC**	**Sensitivity**	**Specificity**	**Number of biomarkers**
Ensemble Boosting with random undersampling	0.937	0.855	0.855	5
Ensemble Boosting	0.905	0.855	0.630	5
SVM	0.624	0.834	0.414	5
MFNN with 1 hidden layer	0.888	0.830	0.571	5
MFNN with 2 hidden layers	0.884	0.834	0.625	5
MFNN with 3 hidden layers	0.863	0.828	0.615	5
Logistic Regression	0.864	0.848	0.567	5
Random Forests	0.851	0.823	0.420	5
naive Bayes	0.849	0.834	0.669	5
C4.5 decision tree	0.732	0.844	0.442	5

AUC = the area under the receiver operating characteristic curve; DAO = D-amino acid oxidase; G72 (also known as DAOA) = D-amino acid oxidase activator; MFNNs = multilayer feedforward neural networks; SVM = support vector machine.

Table S6. The results of leave-one-out cross-validation experiments for differentiating schizophrenia patients from healthy individuals using ensemble boosting with random undersampling, ensemble boosting, SVM, MFNNs, logistic regression, random forests, naive Bayes, and C4.5 decision tree with DAO protein levels.

Algorithm	AUC	Sensitivity	Specificity	Number of biomarkers
Ensemble Boosting with random undersampling	0.603	0.541	0.541	1
Ensemble Boosting	0.602	0.800	0.194	1
SVM	0.500	0.805	0.195	1
MFNN with 1 hidden layer	0.694	0.805	0.195	1
MFNN with 2 hidden layers	0.672	0.805	0.195	1
MFNN with 3 hidden layers	0.449	0.805	0.195	1
Logistic Regression	0.698	0.805	0.221	1
Random Forests	0.578	0.667	0.302	1
naive Bayes	0.674	0.805	0.195	1
C4.5 decision tree	0.500	0.805	0.195	1

AUC = the area under the receiver operating characteristic curve; DAO = D-amino acid oxidase; MFNNs = multilayer feedforward neural networks; SVM = support vector machine.

Table S7. The results of leave-one-out cross-validation experiments for differentiating schizophrenia patients from healthy individuals using ensemble boosting with random undersampling, ensemble boosting, SVM, MFNNs, logistic regression, random forests, naive Bayes, and C4.5 decision tree with G72 protein levels.

**Algorithm**	**AUC**	**Sensitivity**	**Specificity**	**Number of biomarkers**
Ensemble Boosting with random undersampling	0.610	0.727	0.727	1
Ensemble Boosting	0.753	0.805	0.239	1
SVM	0.500	0.805	0.195	1
MFNN with 1 hidden layer	0.796	0.805	0.283	1
MFNN with 2 hidden layers	0.778	0.805	0.195	1
MFNN with 3 hidden layers	0.449	0.805	0.195	1
Logistic Regression	0.799	0.794	0.324	1
Random Forests	0.724	0.769	0.398	1
naive Bayes	0.771	0.805	0.195	1
C4.5 decision tree	0.500	0.805	0.195	1

AUC = the area under the receiver operating characteristic curve; G72 (also known as DAOA) = D-amino acid oxidase activator; MFNNs = multilayer feedforward neural networks; SVM = support vector machine.

Table S8. The results of leave-one-out cross-validation experiments for differentiating schizophrenia patients from healthy individuals using ensemble boosting with random undersampling, ensemble boosting, SVM, MFNNs, logistic regression, random forests, naive Bayes, and C4.5 decision tree with melatonin protein levels.

Algorithm	AUC	Sensitivity	Specificity	Number of biomarkers
Ensemble Boosting with random undersampling	0.826	0.767	0.767	1
Ensemble Boosting	0.784	0.803	0.344	1
SVM	0.620	0.834	0.405	1
MFNN with 1 hidden layer	0.788	0.834	0.458	1
MFNN with 2 hidden layers	0.783	0.839	0.459	1
MFNN with 3 hidden layers	0.748	0.844	0.442	1
Logistic Regression	0.658	0.825	0.306	1
Random Forests	0.547	0.800	0.344	1
naive Bayes	0.656	0.805	0.195	1
C4.5 decision tree	0.500	0.805	0.195	1

AUC = the area under the receiver operating characteristic curve; MFNNs = multilayer feedforward neural networks; SVM = support vector machine.

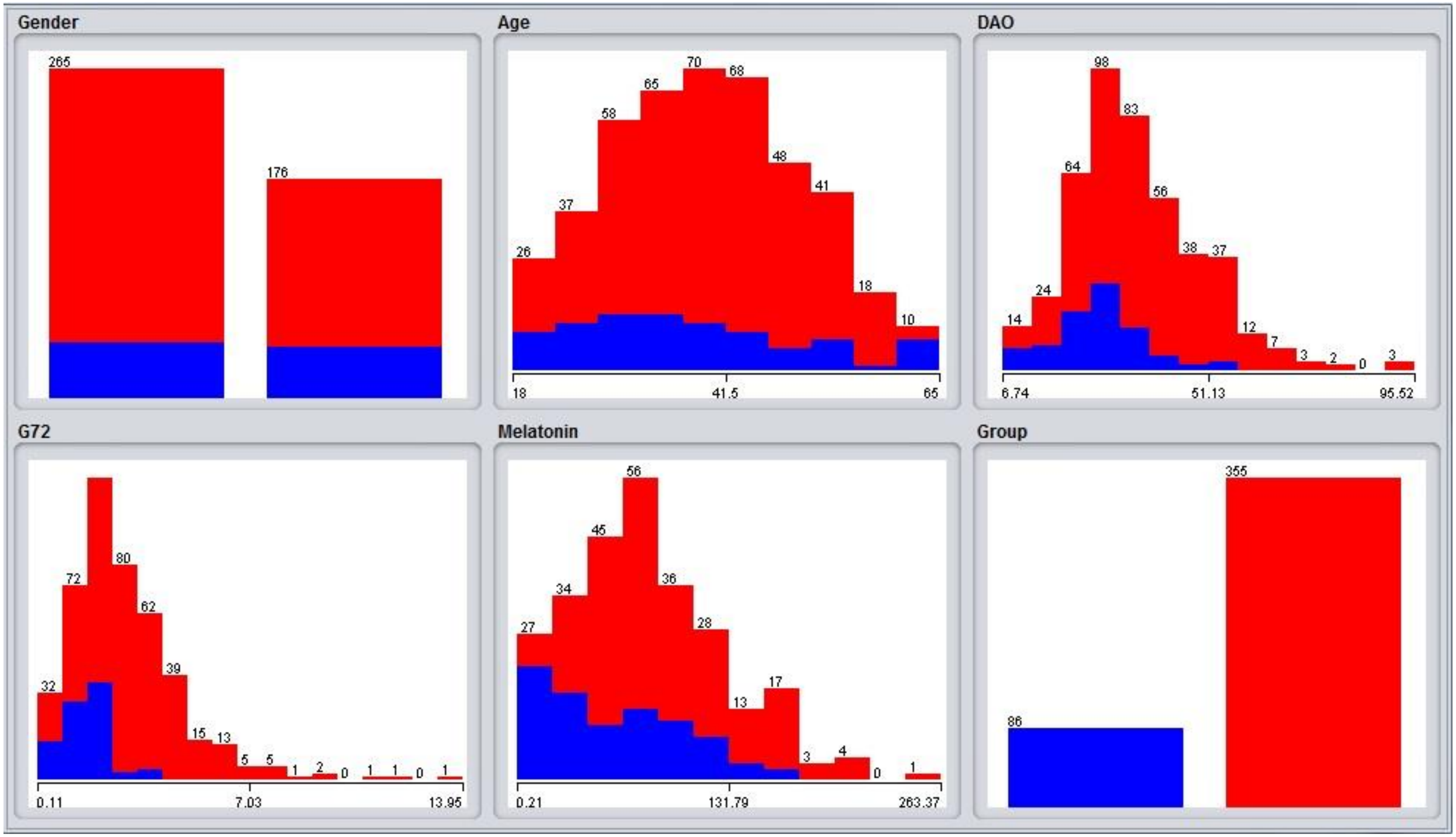

Figure S1. The distribution charts for schizophrenia patients and healthy controls. The upper three charts denote gender, age, and DAO protein levels for schizophrenia patients and healthy controls. The lower three charts denote G72 and melatonin proteins,

and group for schizophrenia patients and healthy controls. The red color represents schizophrenia patients, and the blue color represents healthy controls.

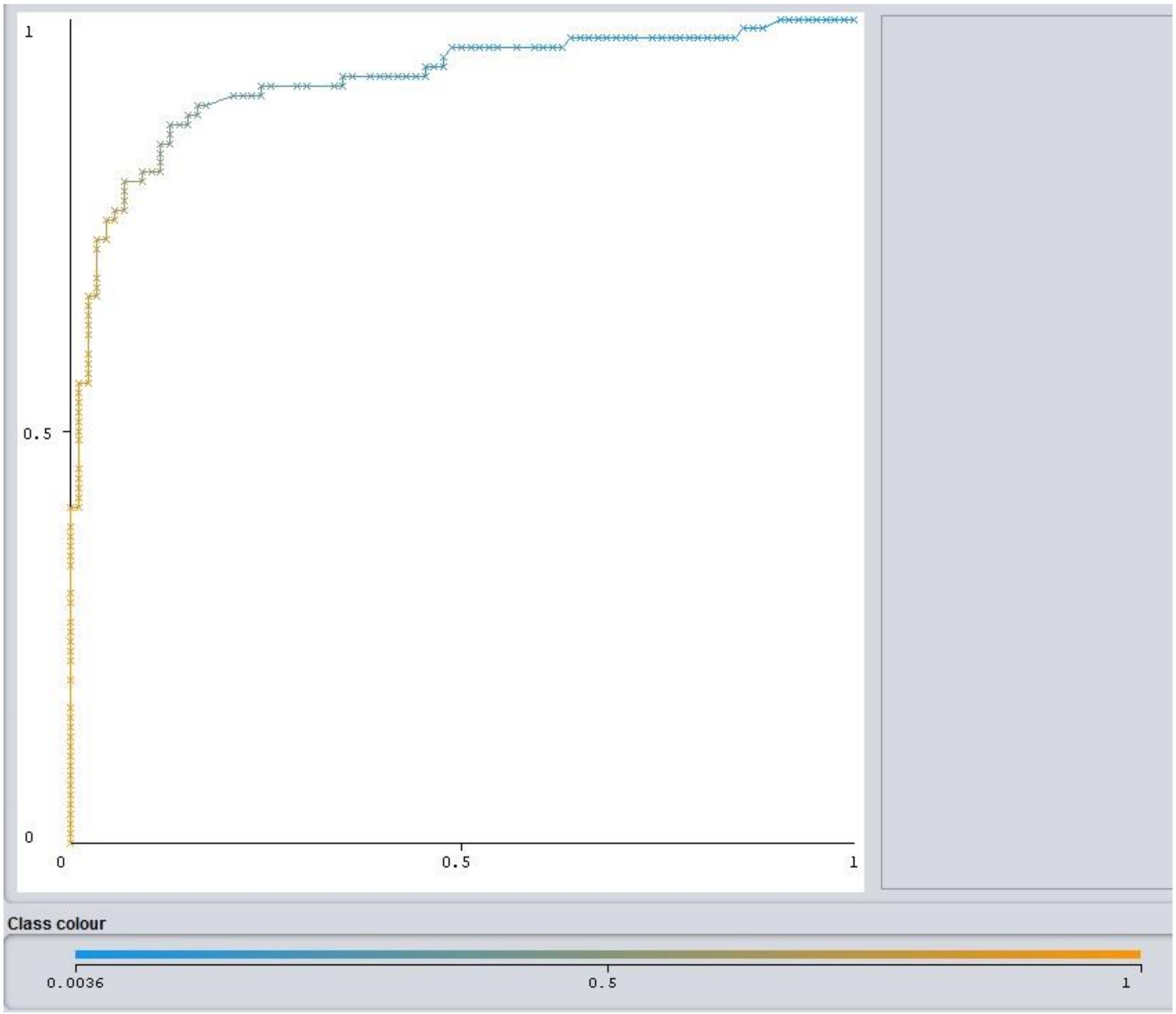

Figure S2. ROC curve plot for ensemble boosting with random undersampling using 5 biomarkers. The vertical axis represents the true positive rate (TPR), and the horizontal axis represents the false positive rate (FPR). ROC = receiver operating characteristic. The "class colour" indicator near the bottom represents the threshold value for each instance. While the darker brown color indicates the higher threshold value, the darker blue color indicates the lower threshold value.

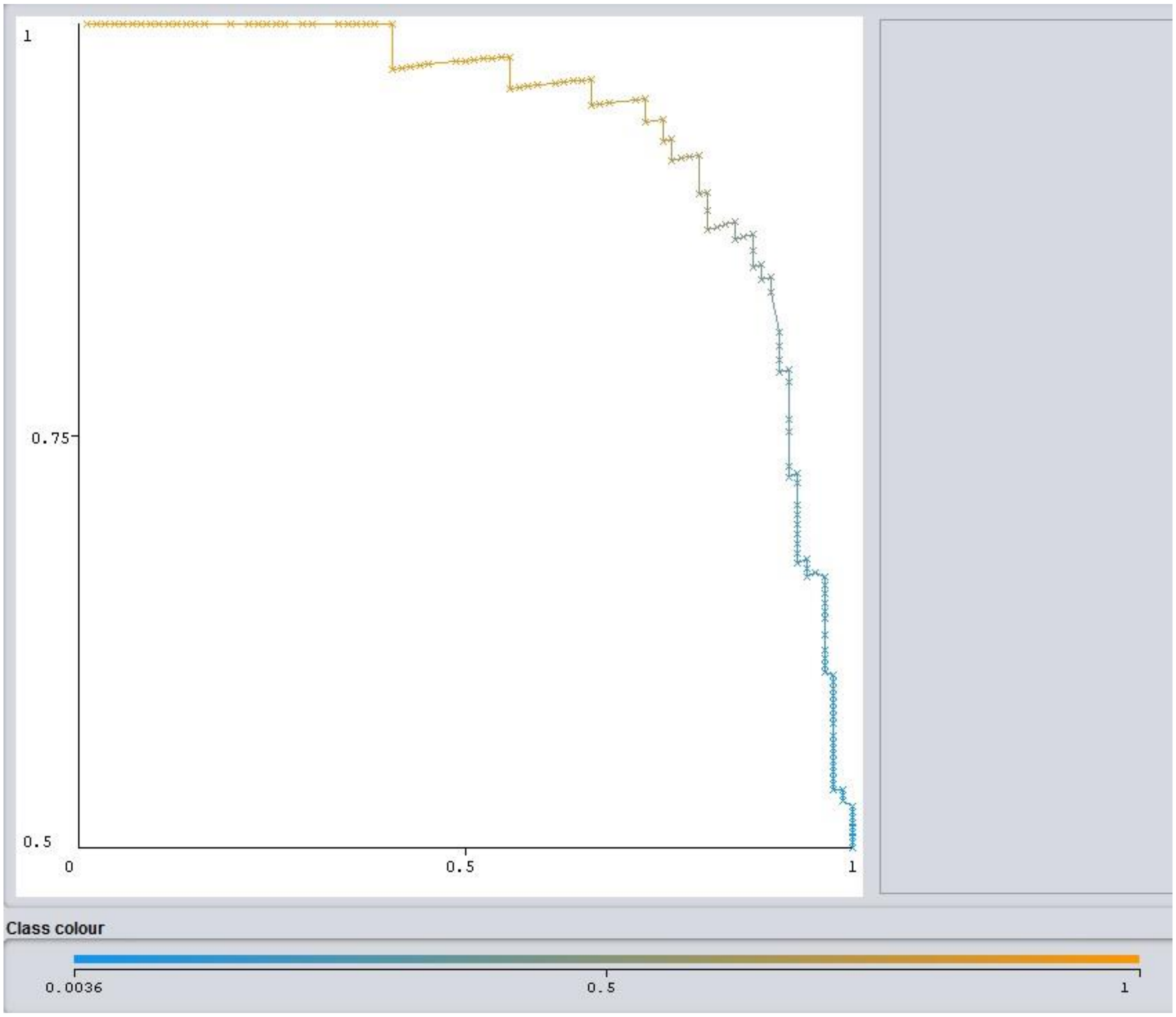

Figure S3. Precision-recall curve plot for ensemble boosting with random undersampling using 5 biomarkers. The vertical axis represents precision, and the horizontal axis represents recall. The "class colour" indicator near the bottom represents the threshold value for each instance. While the darker brown color indicates the higher threshold value, the darker blue color indicates the lower threshold value.

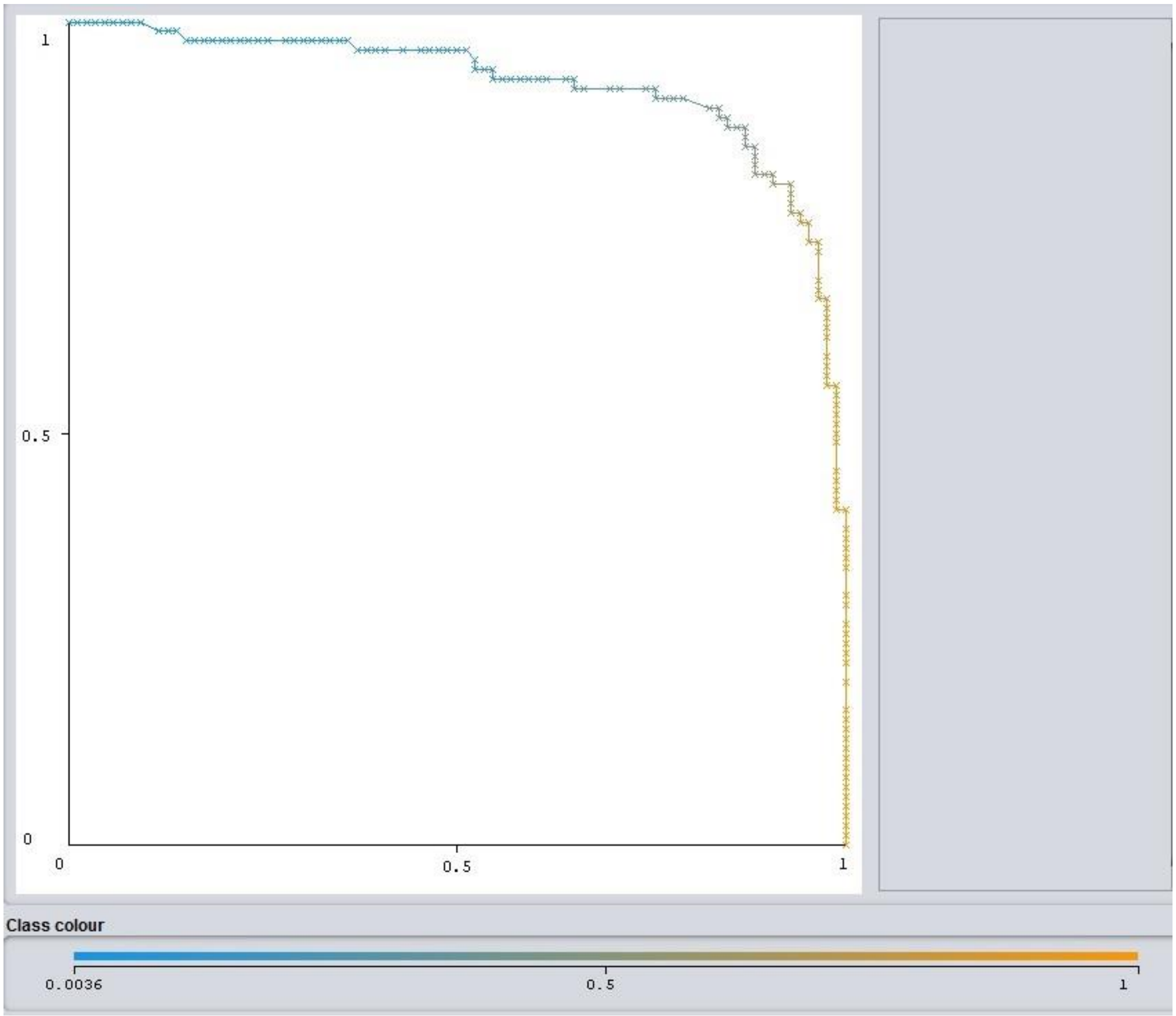

Figure S4. Sensitivity-specificity curve plot for ensemble boosting with random undersampling using 5 biomarkers. The vertical axis represents sensitivity, and the horizontal axis represents specificity. The "class colour" indicator near the bottom represents the threshold value for each instance. While the darker brown color indicates the higher threshold value, the darker blue color indicates the lower threshold value.

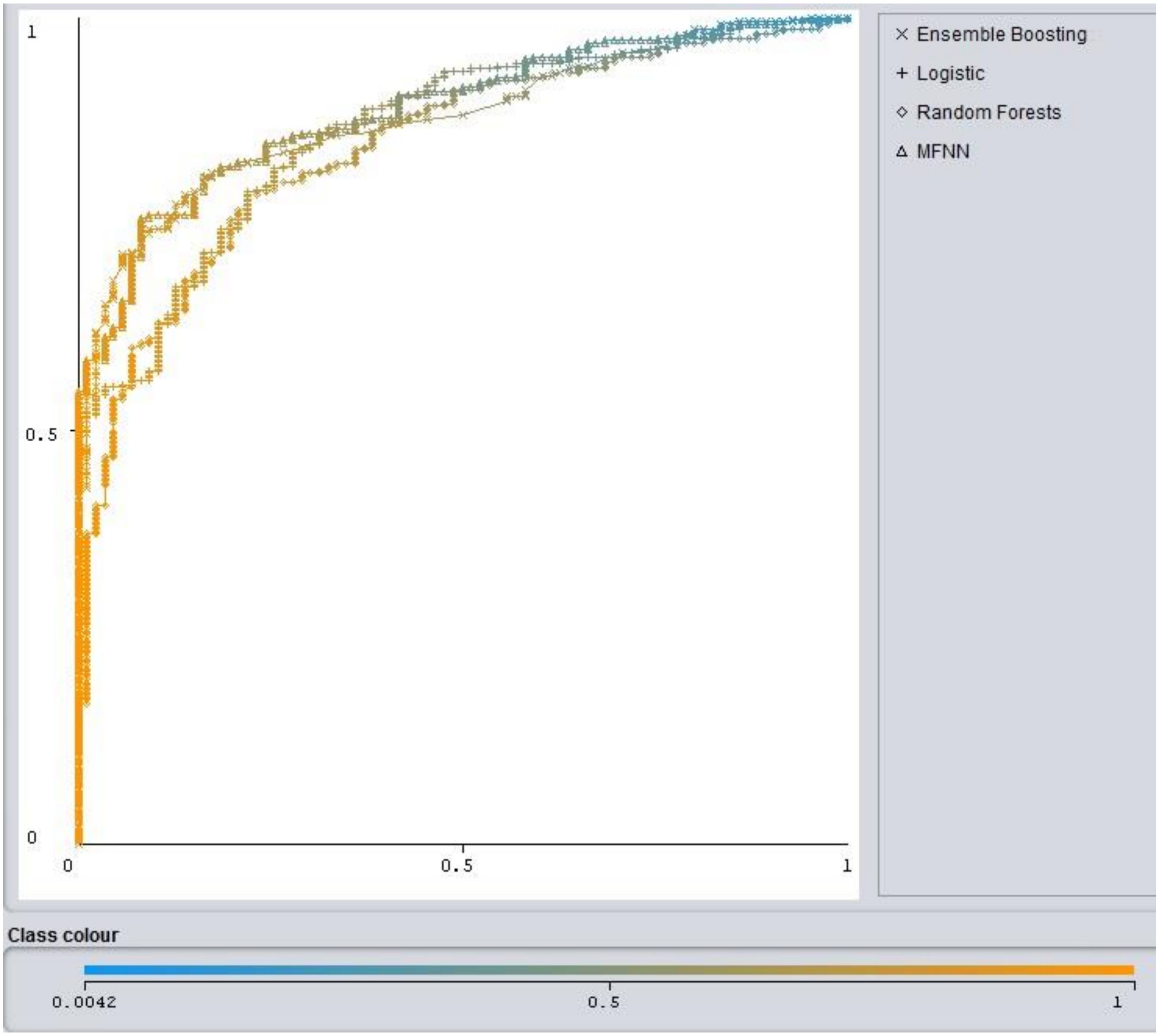

Figure S5. Plot of ROC curves (part A). There are four ROC curves representing ensemble boosting, MFNN (with 2 hidden layers), logistic regression, and random forests using 5 biomarkers. The vertical axis represents the true positive rate (TPR), and the horizontal axis represents the false positive rate (FPR).

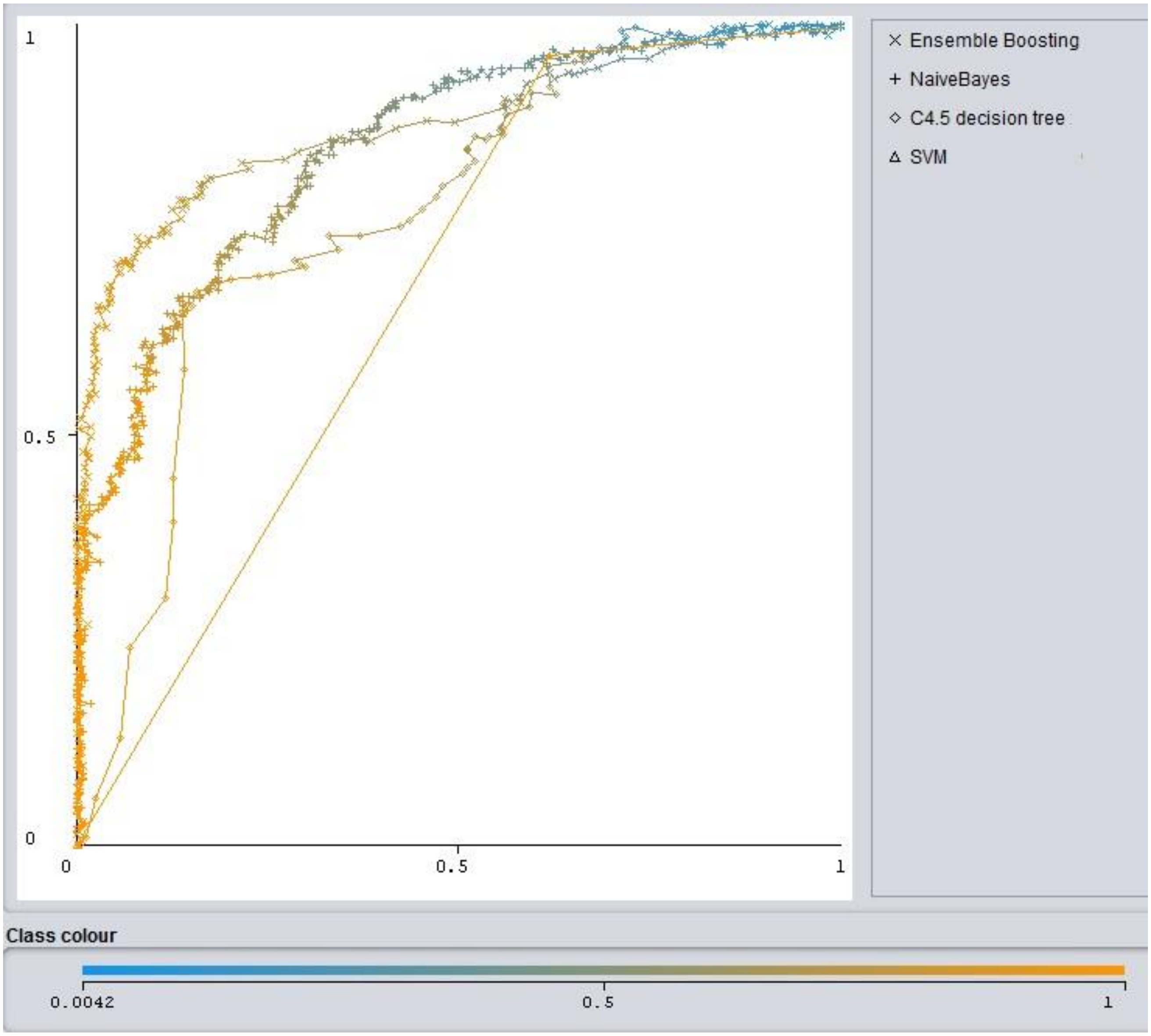

Figure S6. Plot of ROC curves (part B). There are four ROC curves representing ensemble boosting, SVM, naive Bayes, and C4.5 decision tree using 5 biomarkers. The vertical axis represents the true positive rate (TPR), and the horizontal axis represents the false positive rate (FPR).

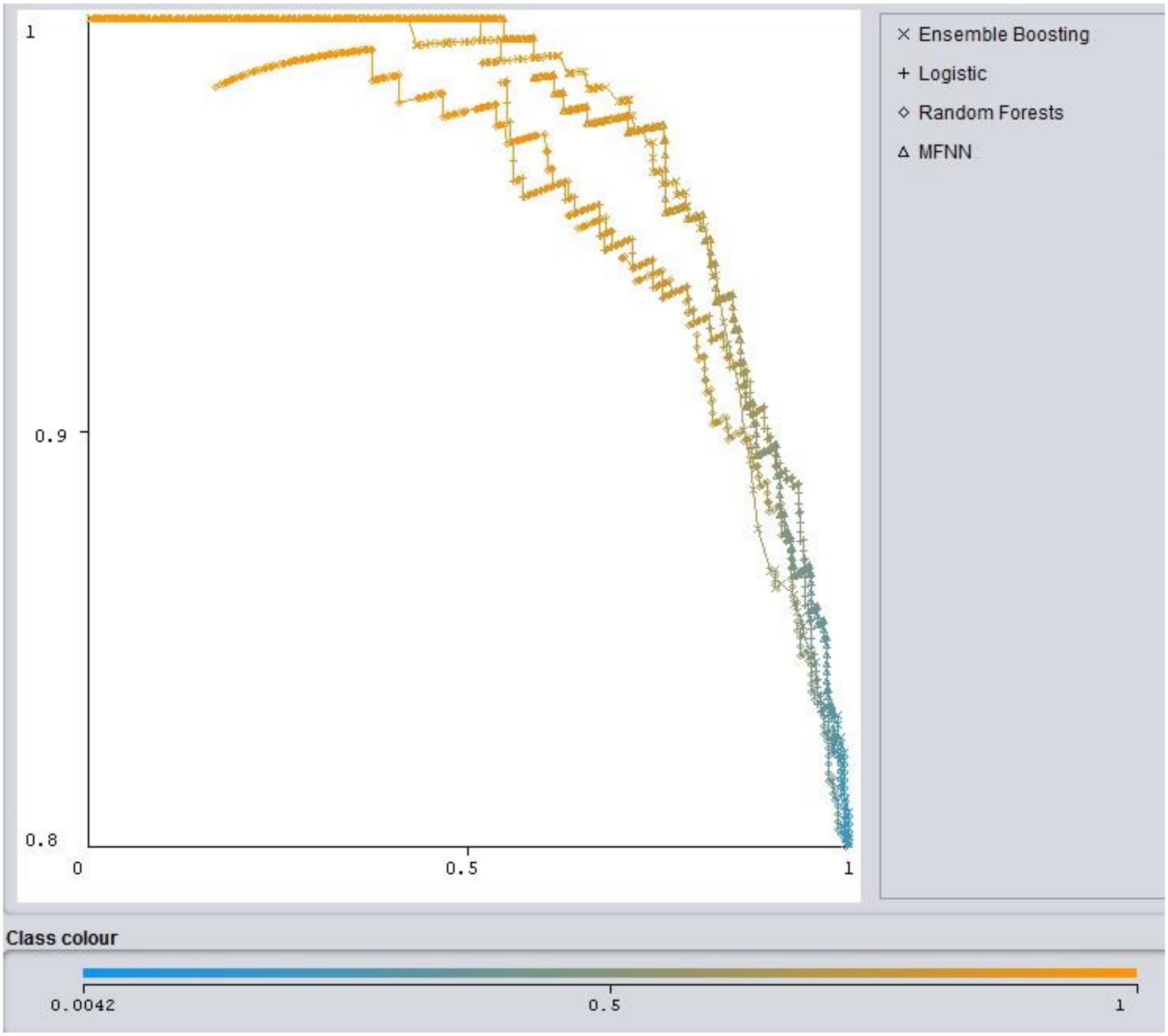

Figure S7. Plot of precision-recall curves (part A). There are four precision-recall curves representing ensemble boosting, MFNN (with 2 hidden layers), logistic regression, and random forests using 5 biomarkers. The vertical axis represents precision, and the horizontal axis represents recall.

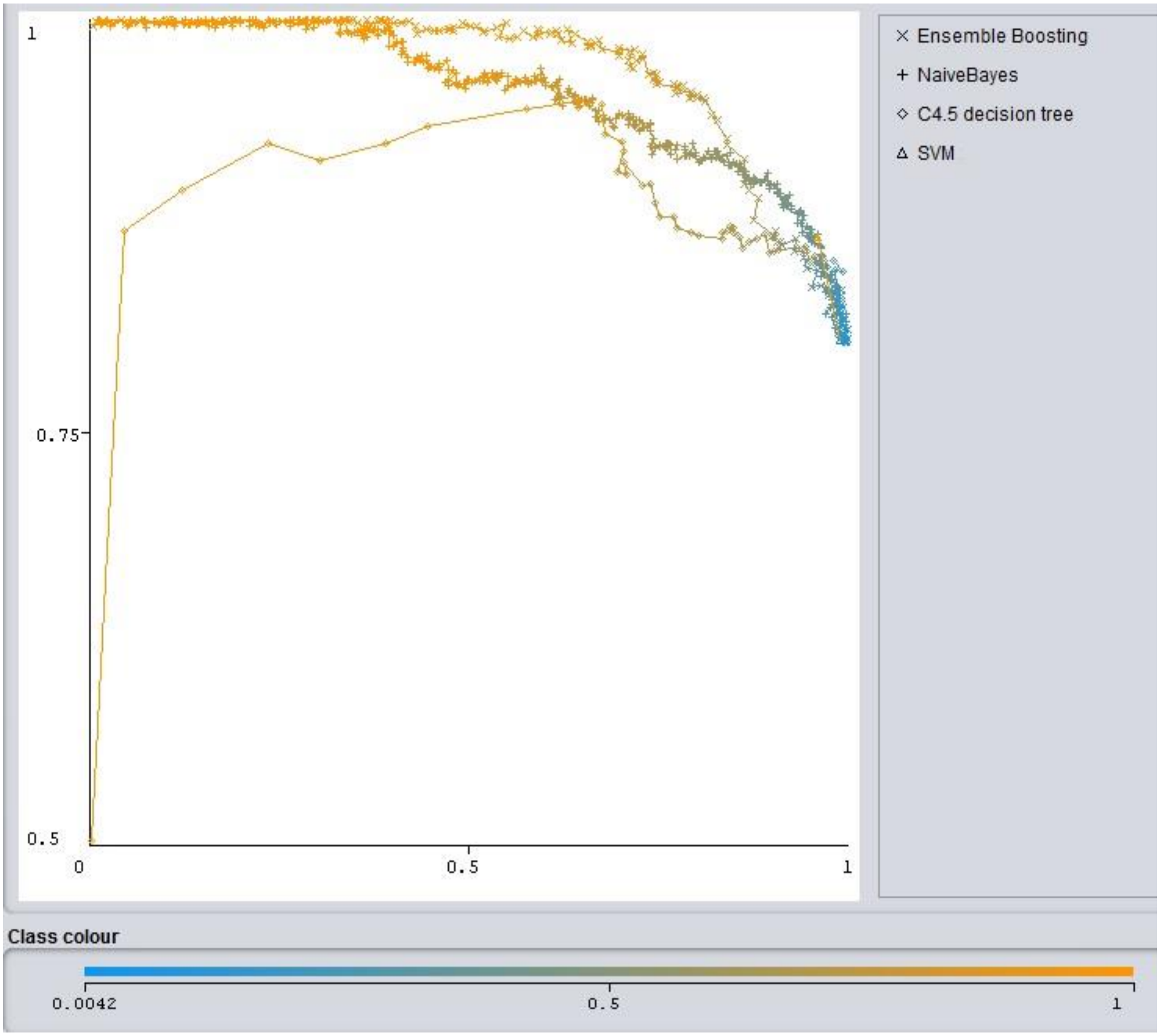

Figure S8. Plot of precision-recall curves (part B). There are four precision-recall curves representing ensemble boosting, SVM, naive Bayes, and C4.5 decision tree using 5 biomarkers. The vertical axis represents precision, and the horizontal axis represents recall.

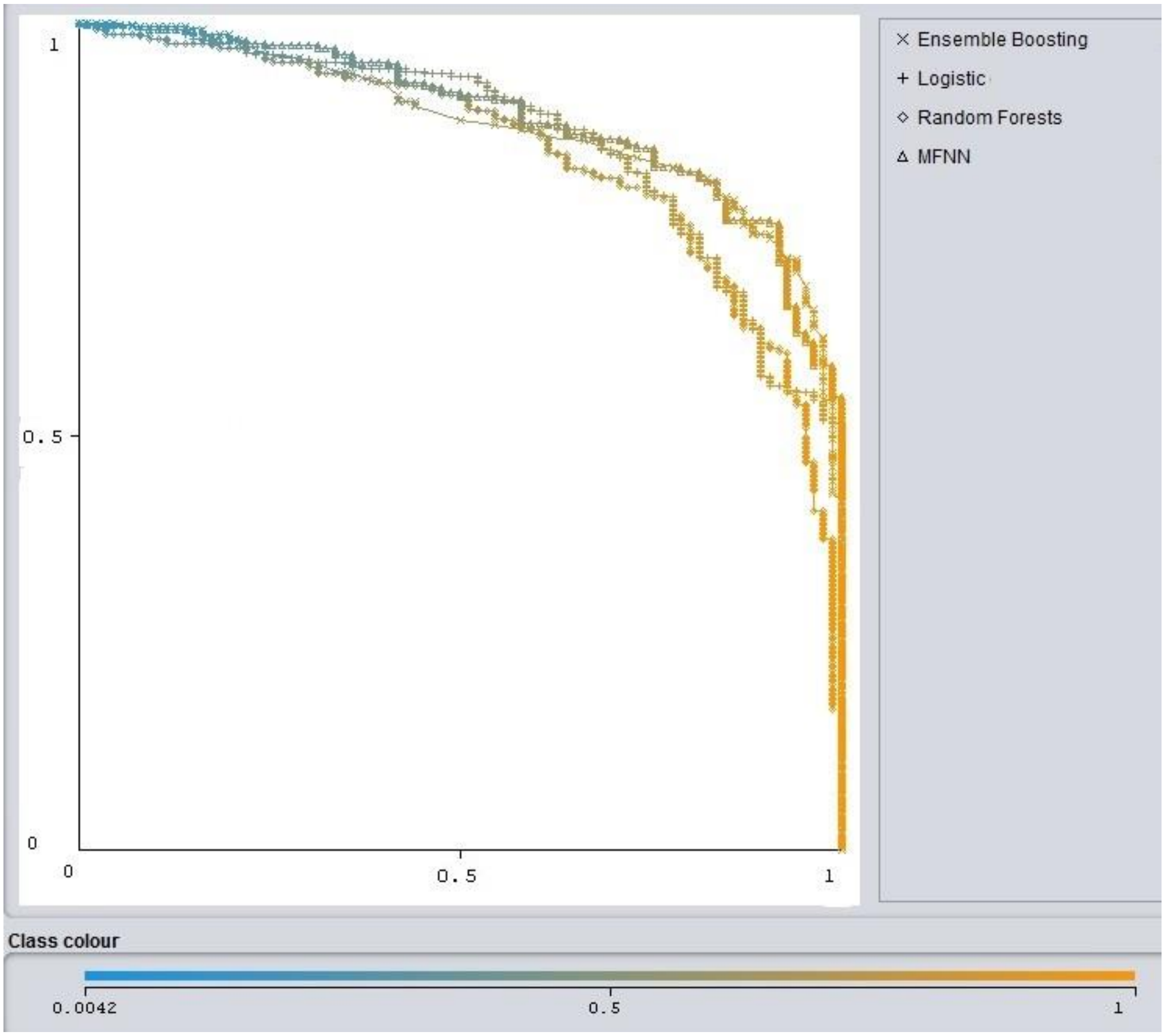

Figure S9. Plot of sensitivity-specificity curves (part A). There are four sensitivity-specificity curves representing ensemble boosting, MFNN (with 2 hidden layers), logistic regression, and random forests using 5 biomarkers. The vertical axis represents sensitivity, and the horizontal axis represents specificity.

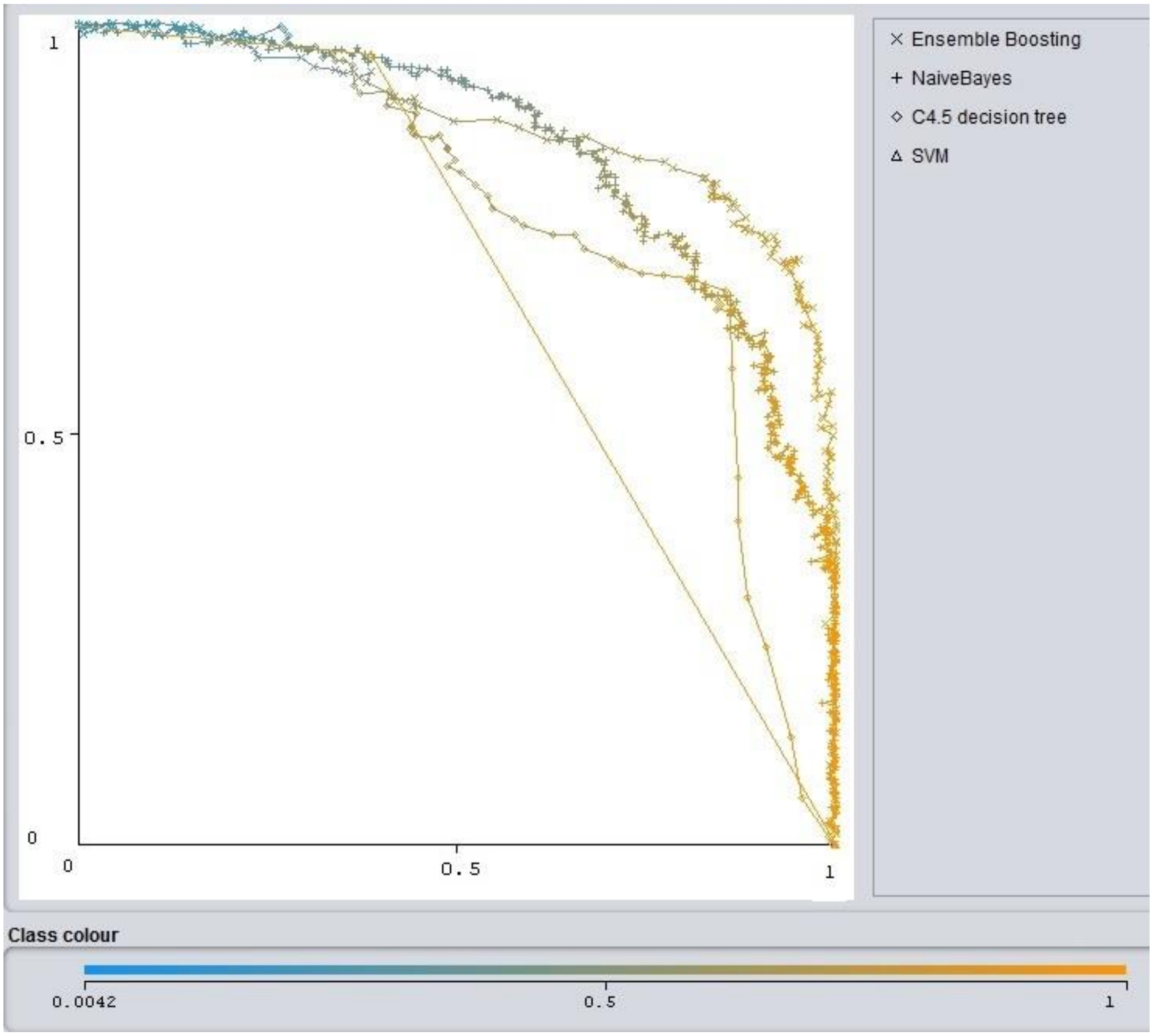

Figure S10. Plot of sensitivity-specificity curves (part B). There are four sensitivity-specificity curves representing ensemble boosting, SVM, naive Bayes, and C4.5 decision tree using 5 biomarkers. The vertical axis represents sensitivity, and the horizontal axis represents specificity.

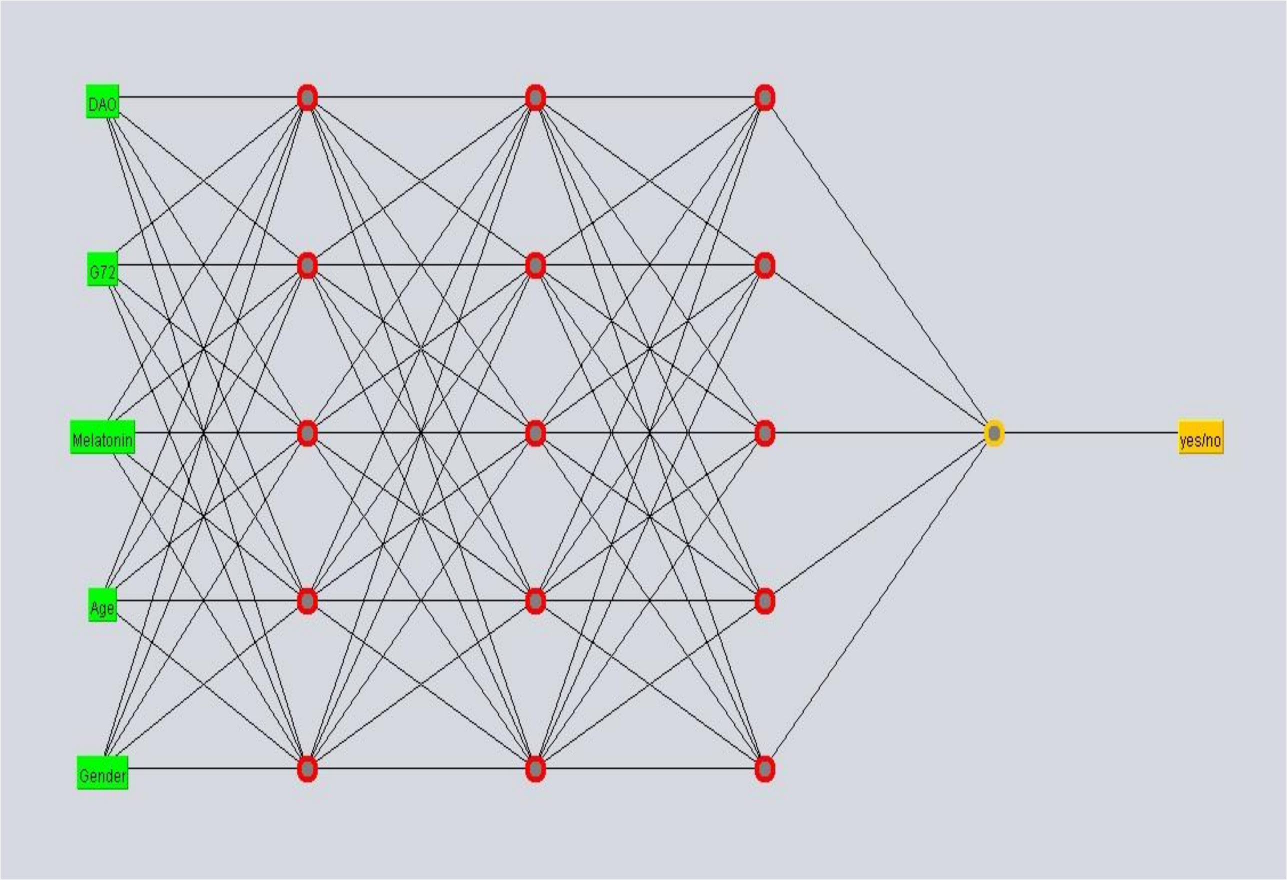

Figure S11. An example architecture of a multilayer feedforward neural network (MFNN) model with 3 hidden layers. The MFNN model contains 5 units in the input layer corresponding to 5 biomarkers (including DAO protein levels, G72 protein levels, melatonin protein levels, age, and gender). The MFNN model is configured with 1 unit in the output layer corresponding to disease outcome (that is, schizophrenia patients or healthy individuals).

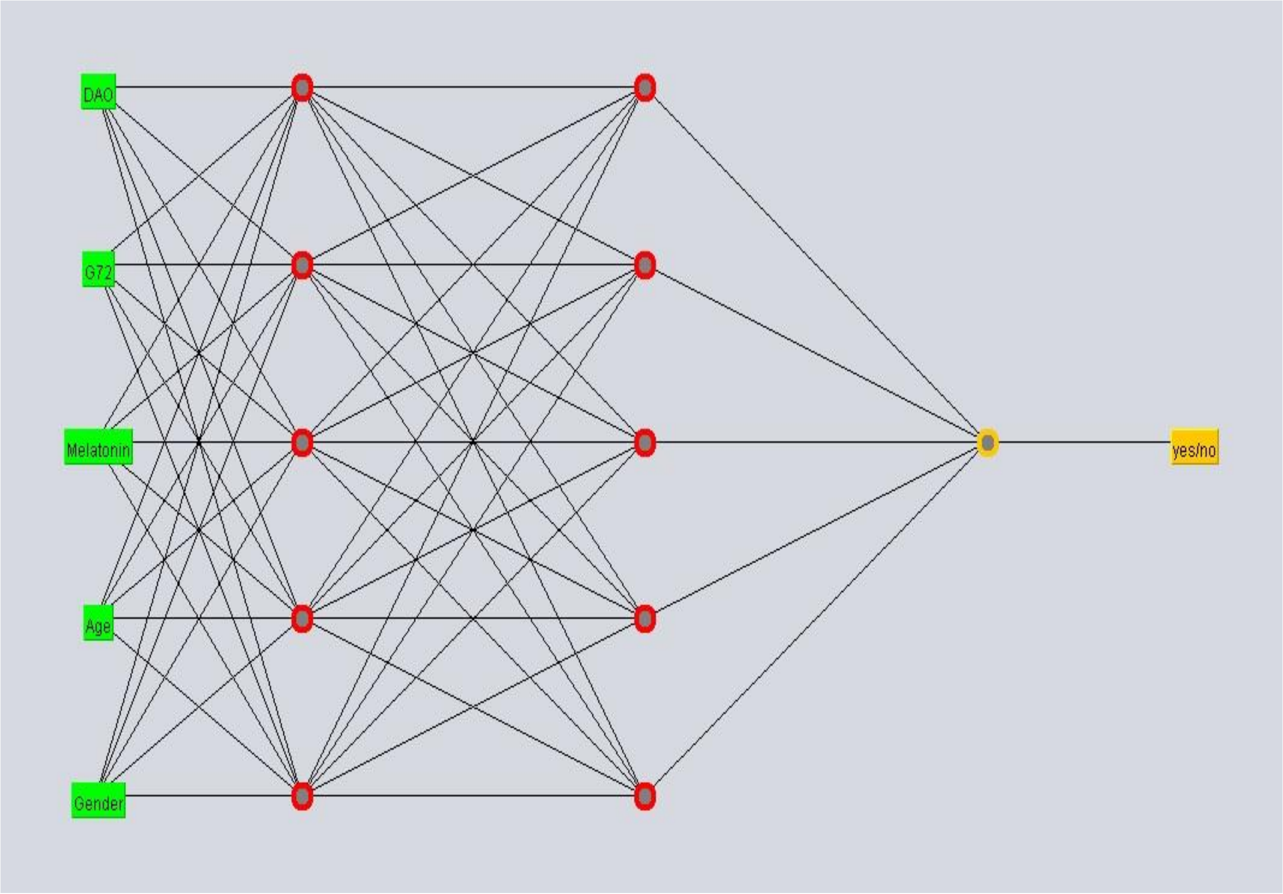

Figure S12. An example architecture of a multilayer feedforward neural network (MFNN) model with 2 hidden layers. The MFNN model contains 5 units in the input layer corresponding to 5 biomarkers (including DAO protein levels, G72 protein levels, melatonin protein levels, age, and gender). The MFNN model is configured with 1 unit in the output layer corresponding to disease outcome (that is, schizophrenia patients or healthy individuals).

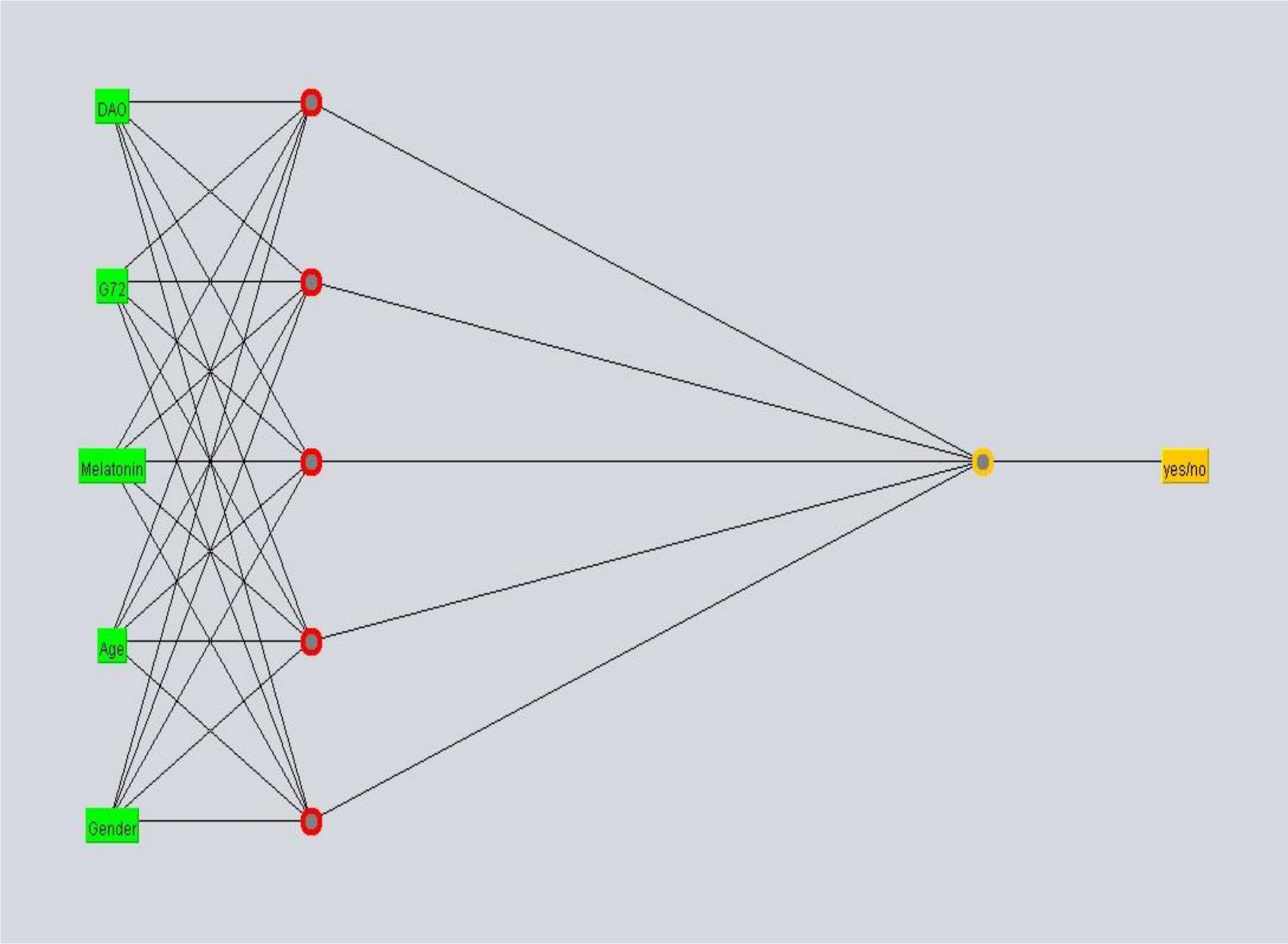

Figure S13. An example architecture of a multilayer feedforward neural network (MFNN) model with 1 hidden layer. The MFNN model contains 5 units in the input layer corresponding to 5 biomarkers (including DAO protein levels, G72 protein levels, melatonin protein levels, age, and gender). The MFNN model is configured with 1 unit in the output layer corresponding to disease outcome (that is, schizophrenia patients or healthy individuals).

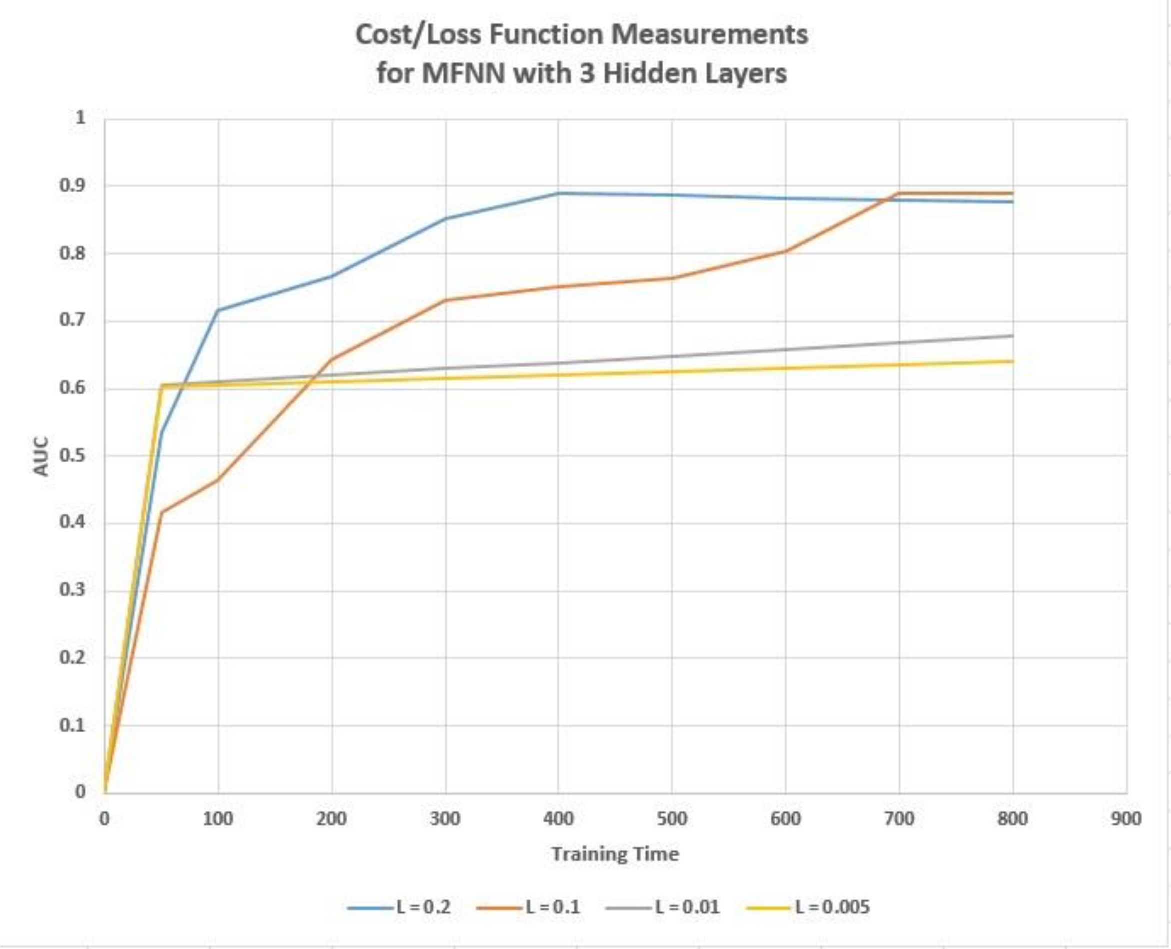

Figure S14. Plot of cost/loss function measurements for a multilayer feedforward neural network (MFNN) model with 3 hidden layers. There are four cost/loss function measurement curves representing learning rates of 0.2, 0.1, 0.01 and 0.005 using 5 biomarkers. The vertical axis represents AUC, and the horizontal axis represents training time. AUC = the area under the ROC curve. ROC = receiver operating characteristic.

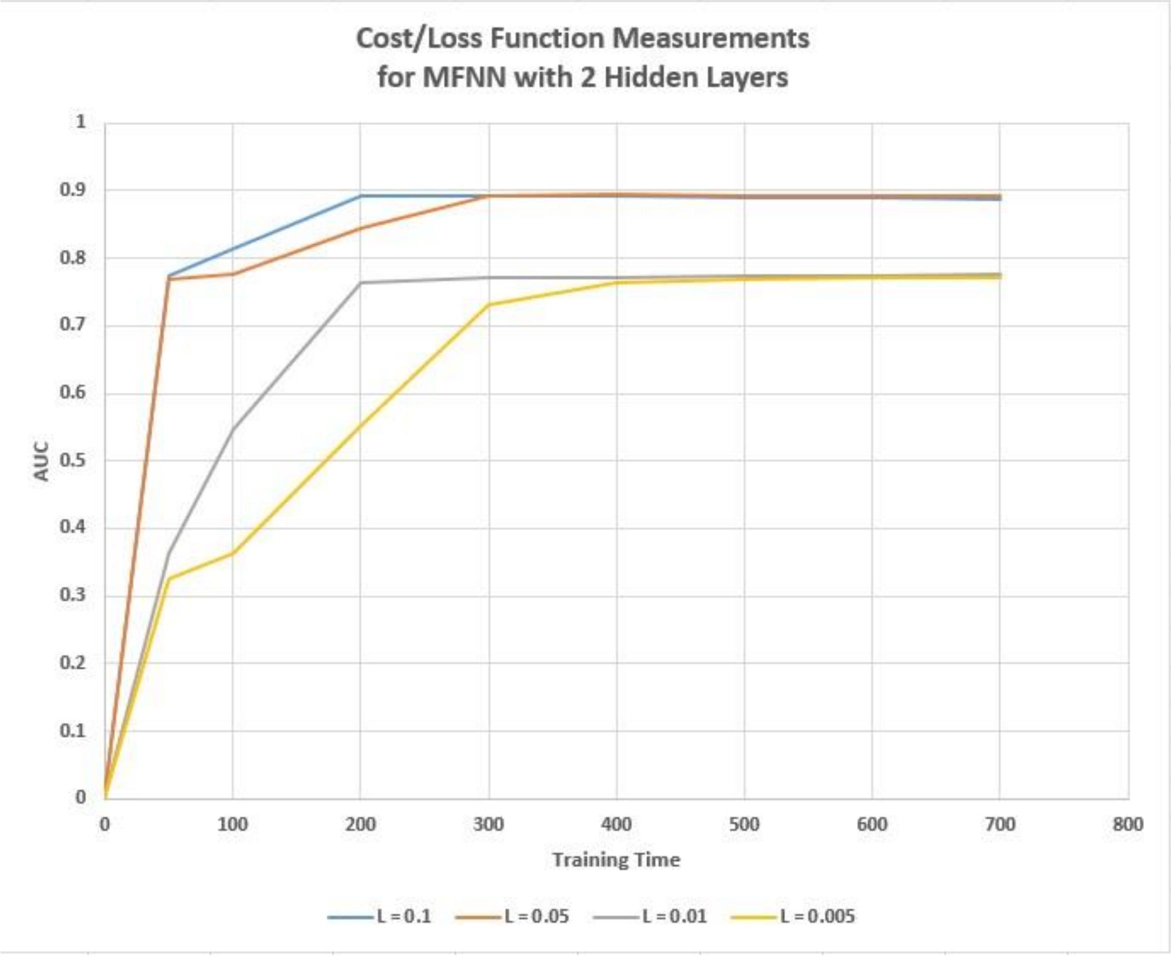

Figure S15. Plot of cost/loss function measurements for a multilayer feedforward neural network (MFNN) model with 2 hidden layers. There are four cost/loss function measurement curves representing learning rates of 0.1, 0.05, 0.01 and 0.005 using 5 biomarkers. The vertical axis represents AUC, and the horizontal axis represents training time. AUC = the area under the ROC curve. ROC = receiver operating characteristic.

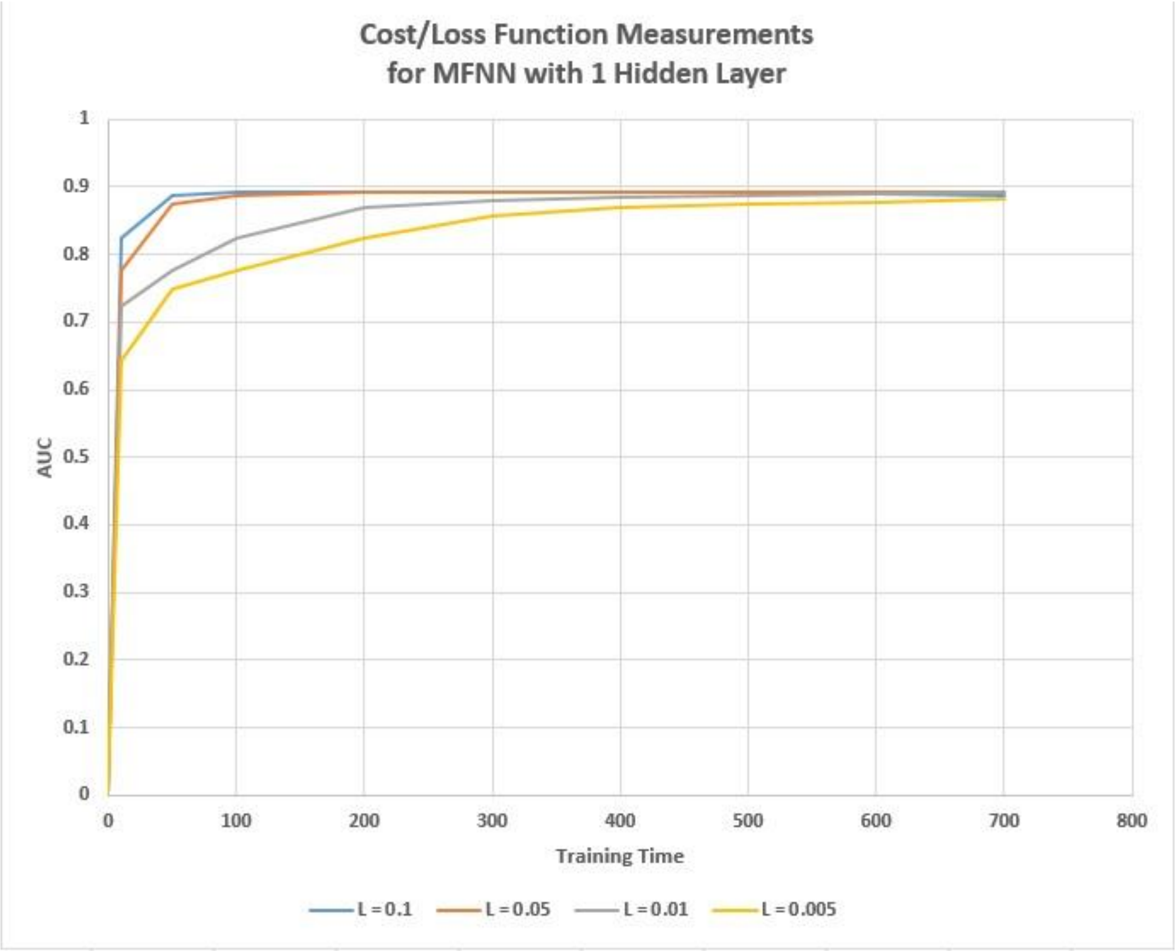

Figure S16. Plot of cost/loss function measurements for a multilayer feedforward neural network (MFNN) model with 1 hidden layer. There are four cost/loss function measurement curves representing learning rates of 0.1, 0.05, 0.01 and 0.005 using 5 biomarkers. The vertical axis represents AUC, and the horizontal axis represents training time. AUC = the area under the ROC curve. ROC = receiver operating characteristic.
